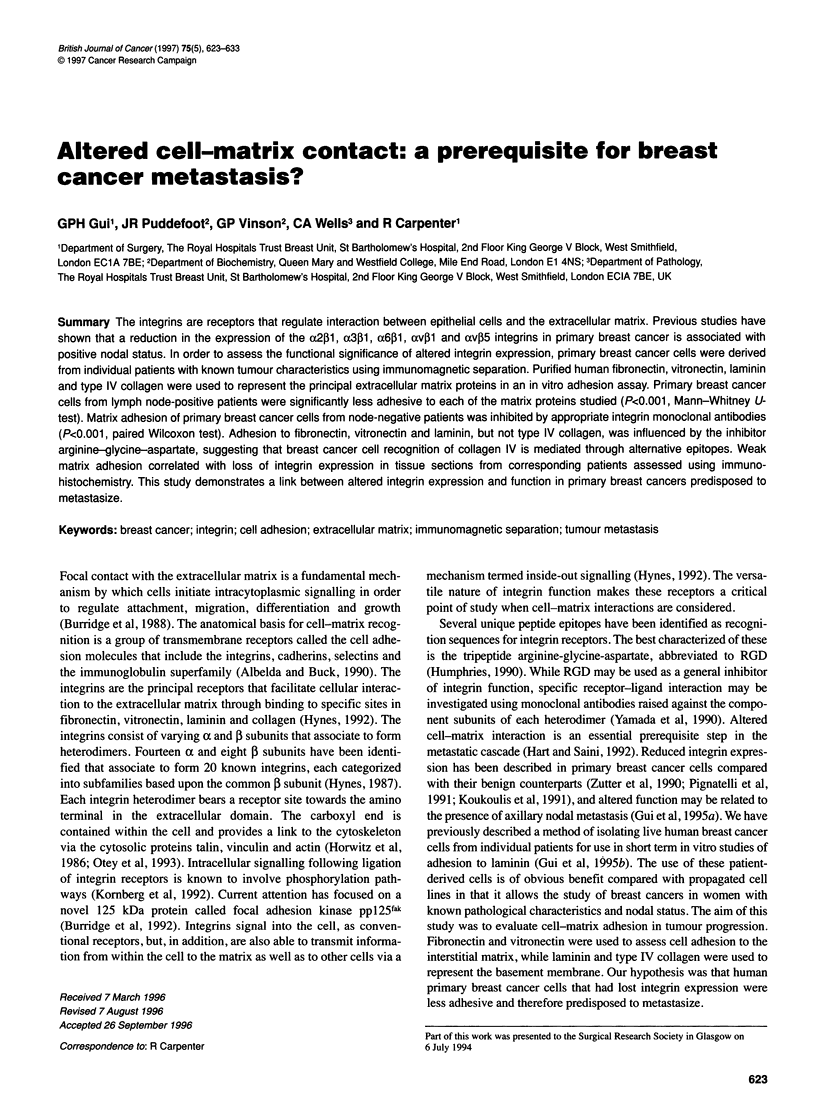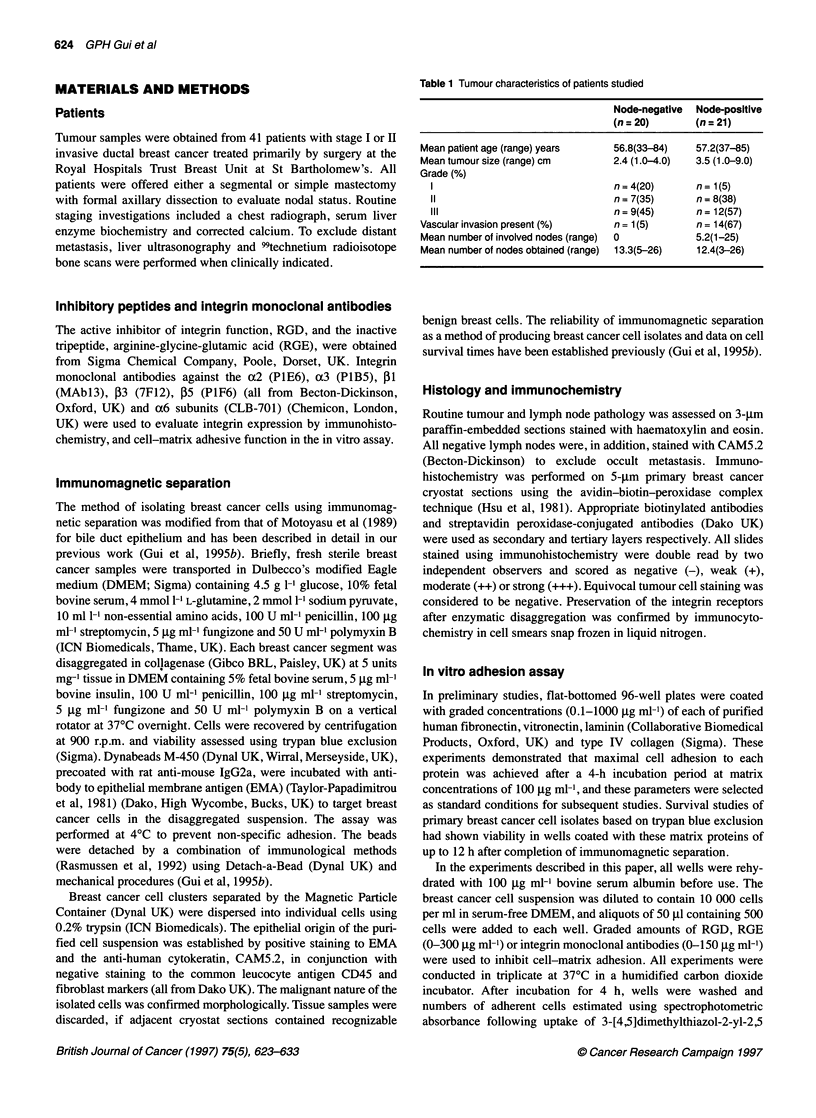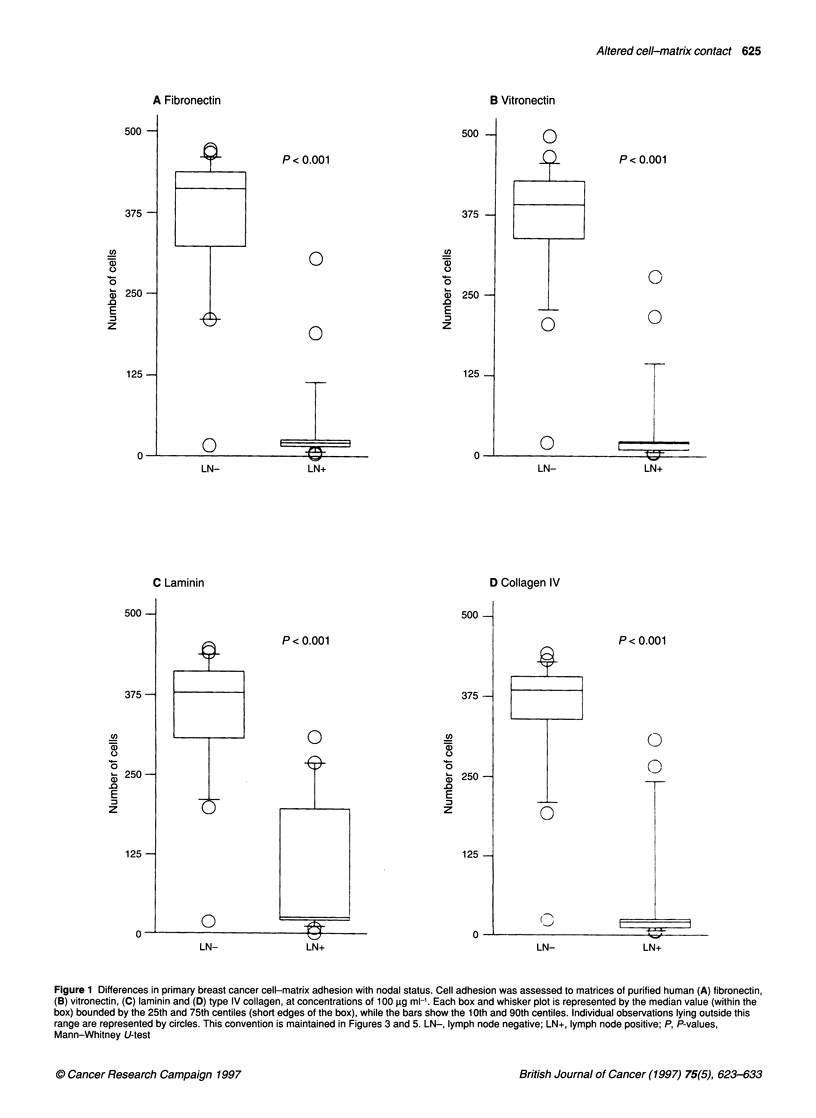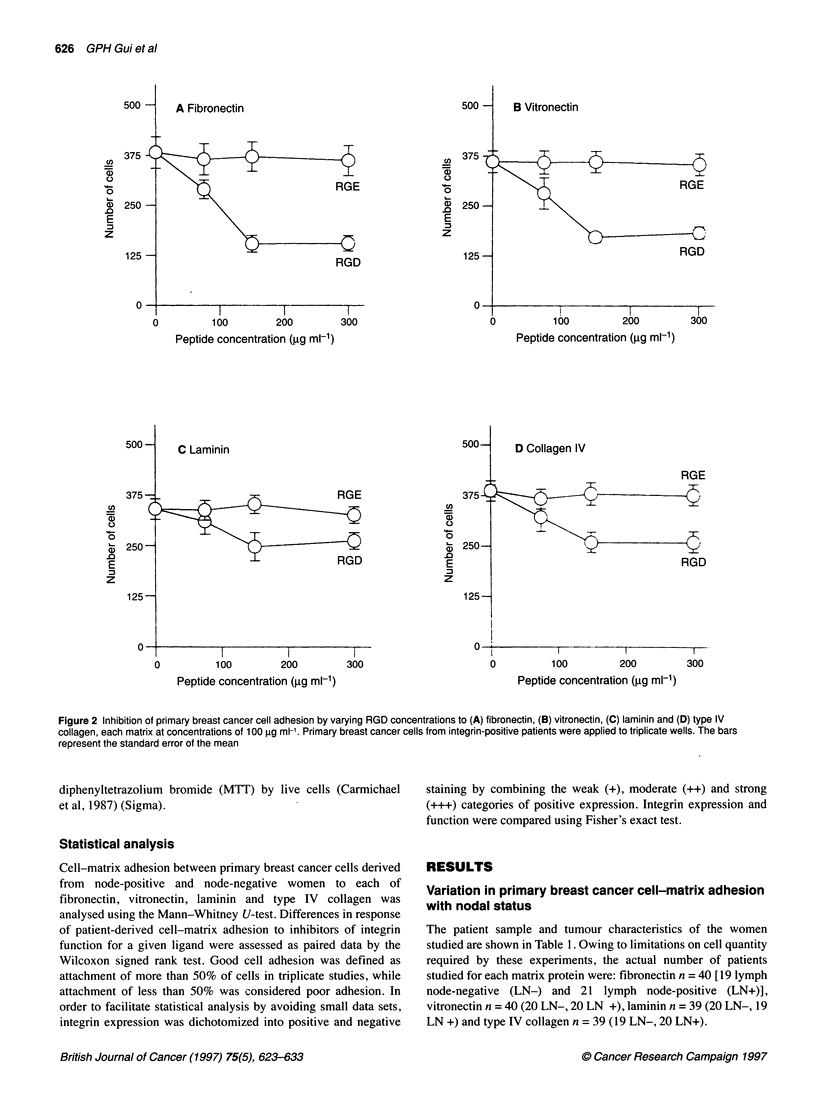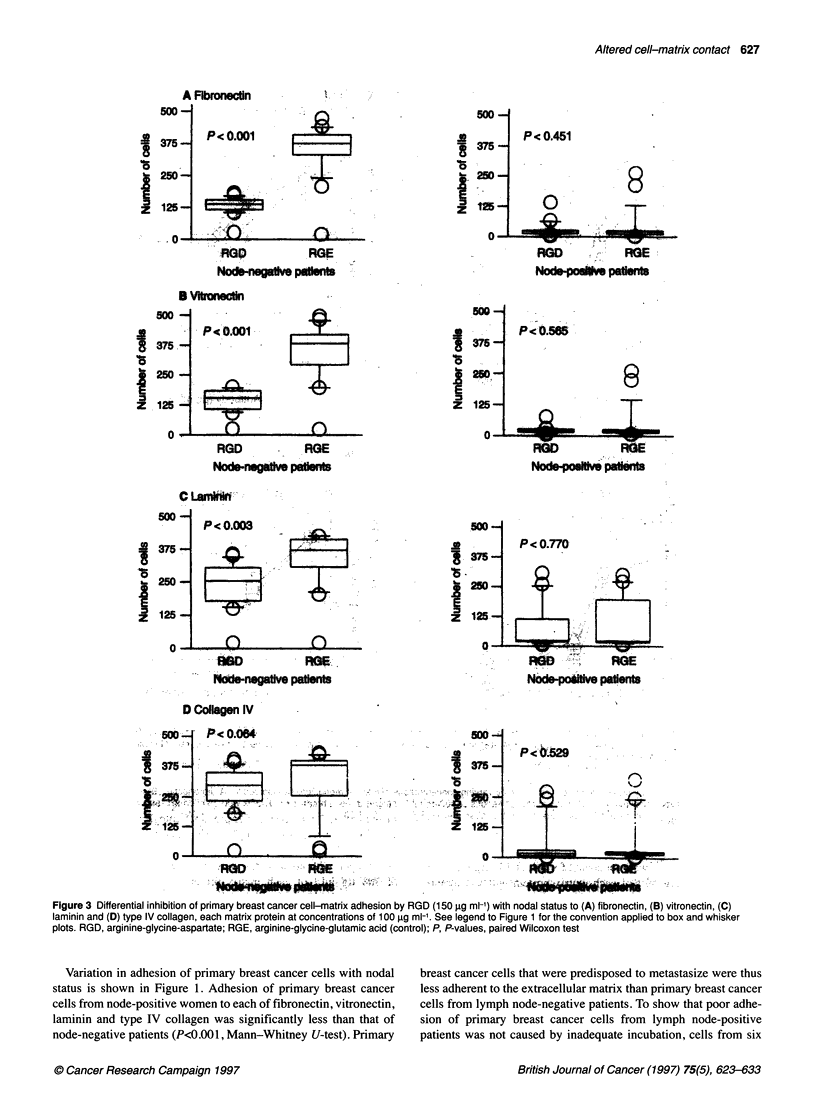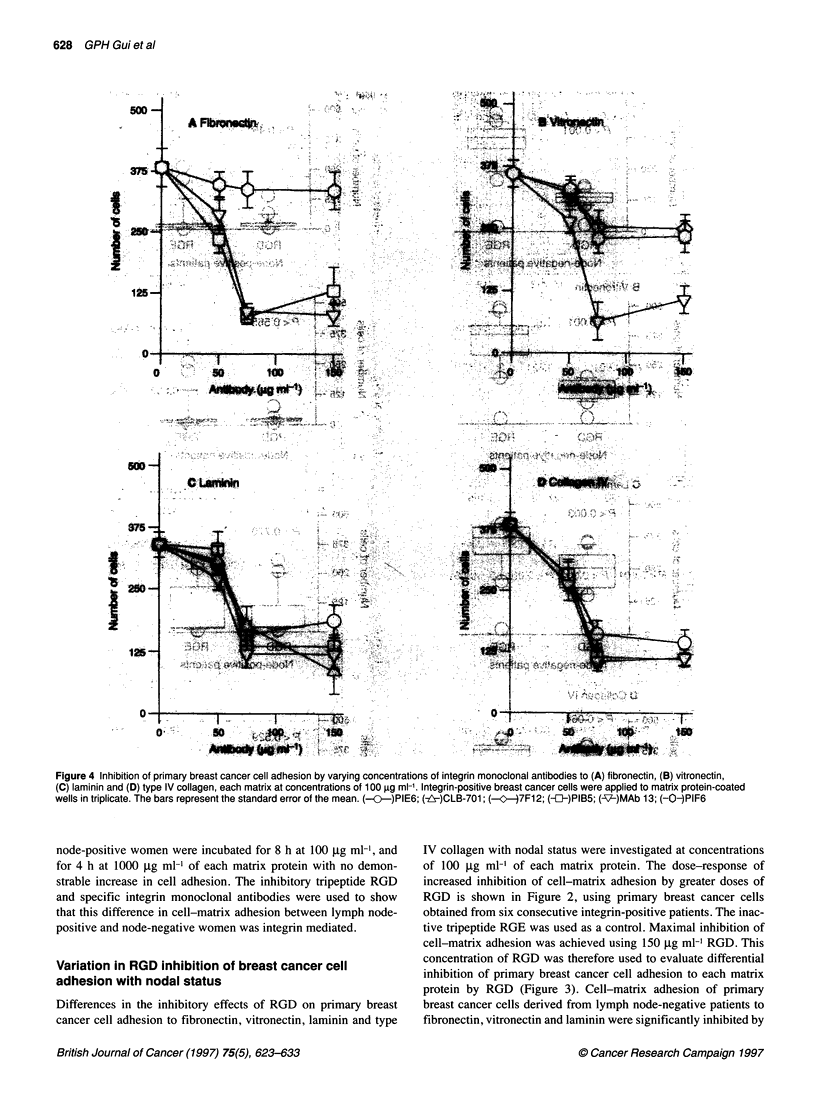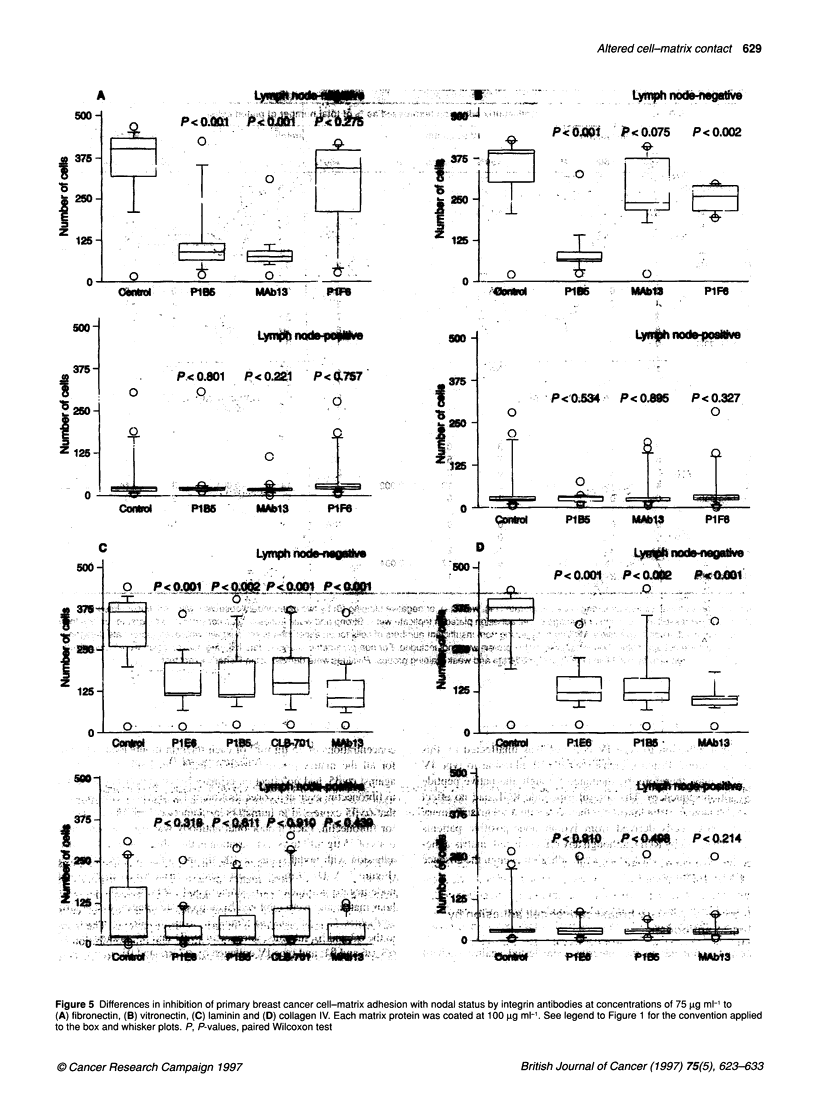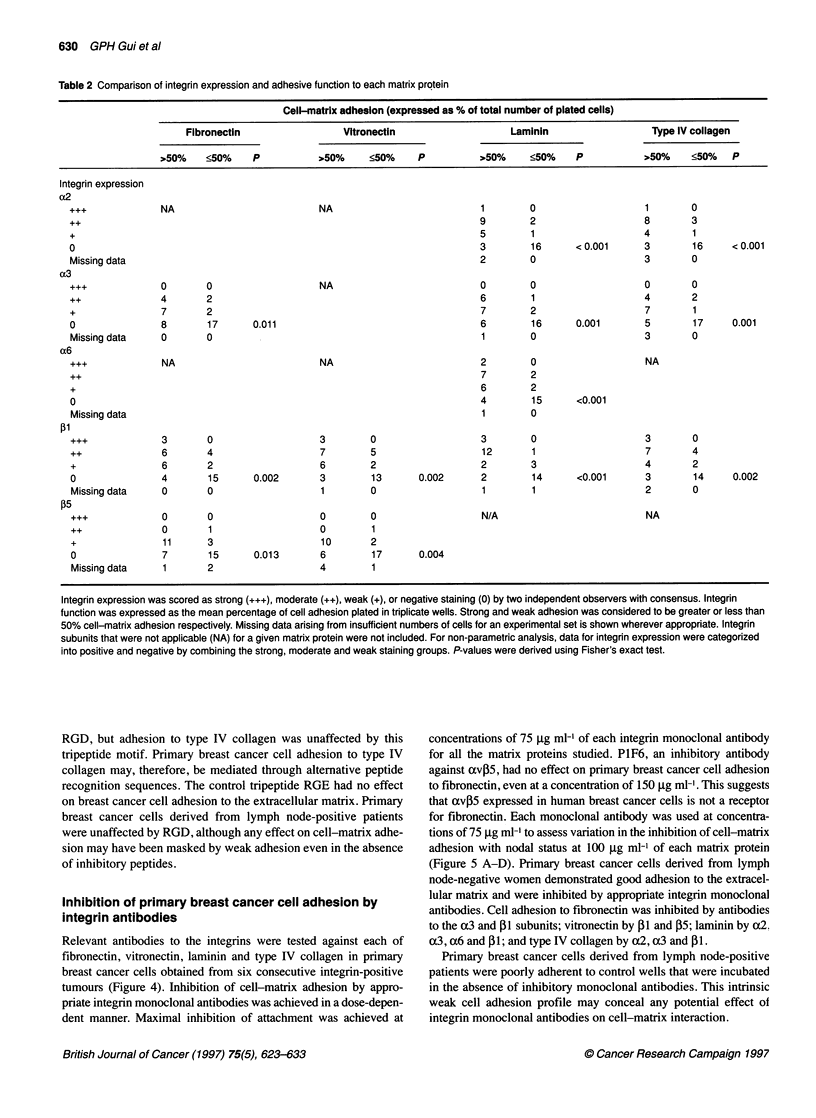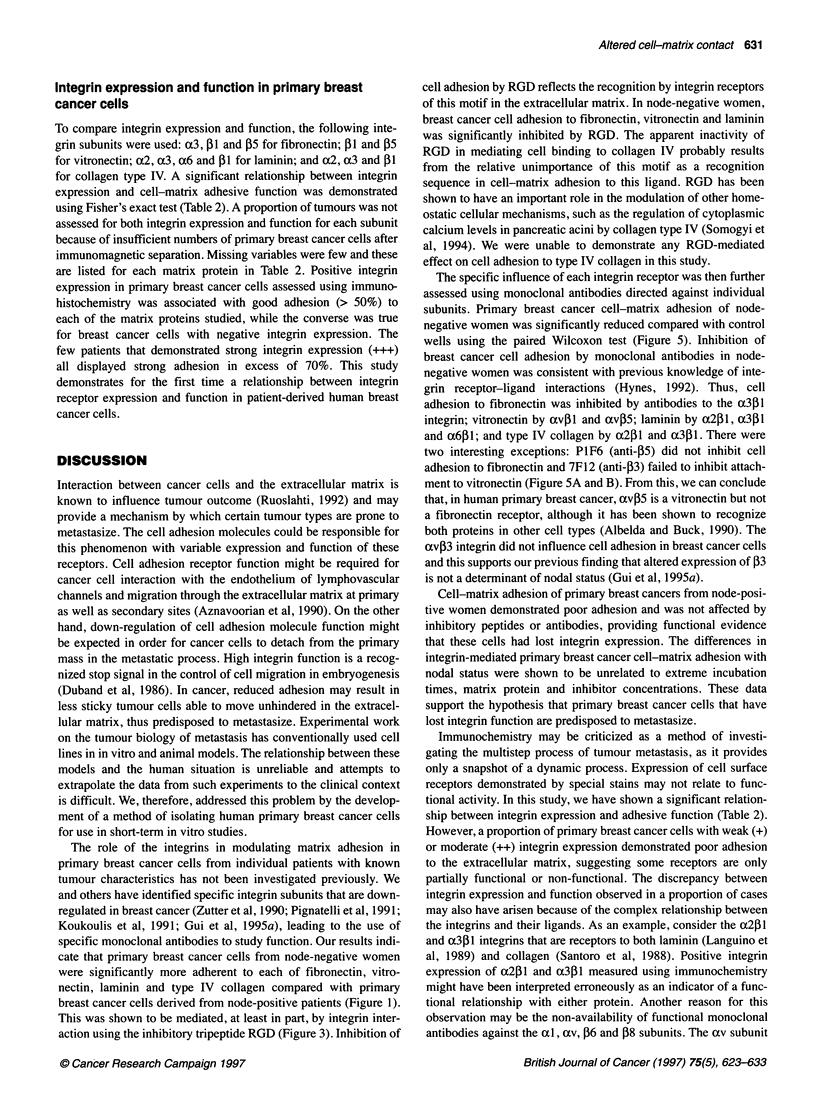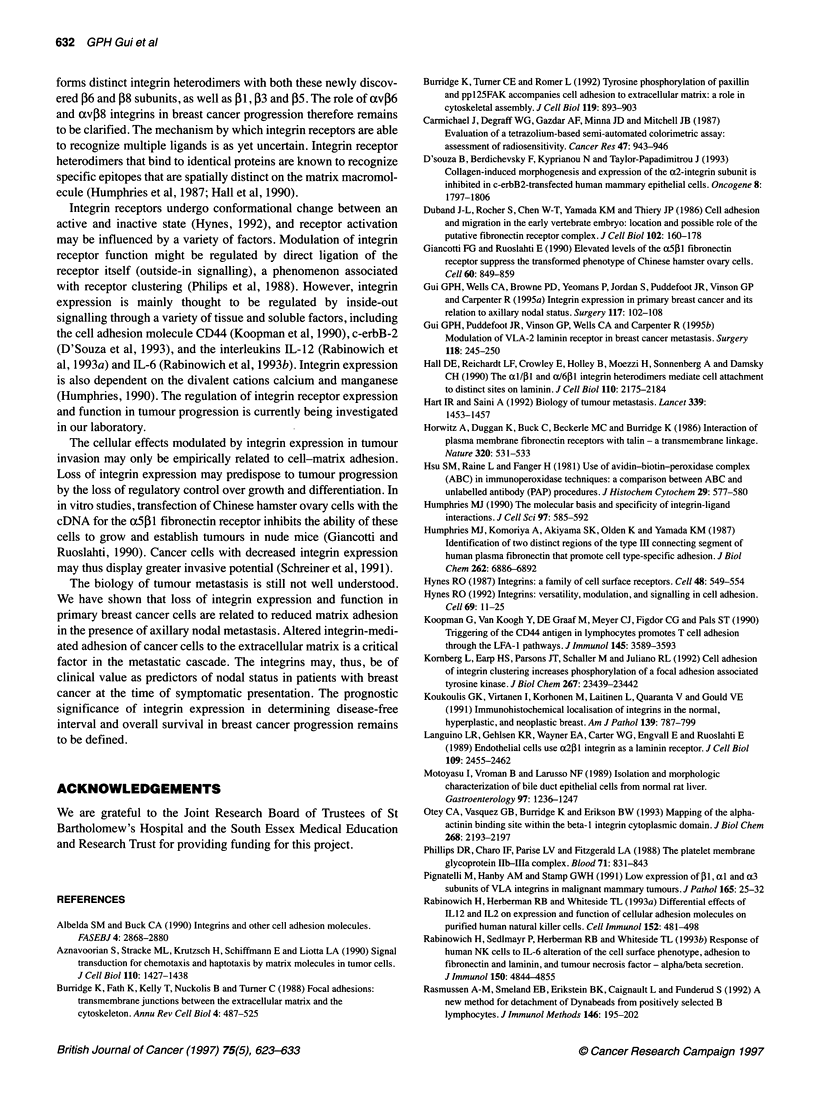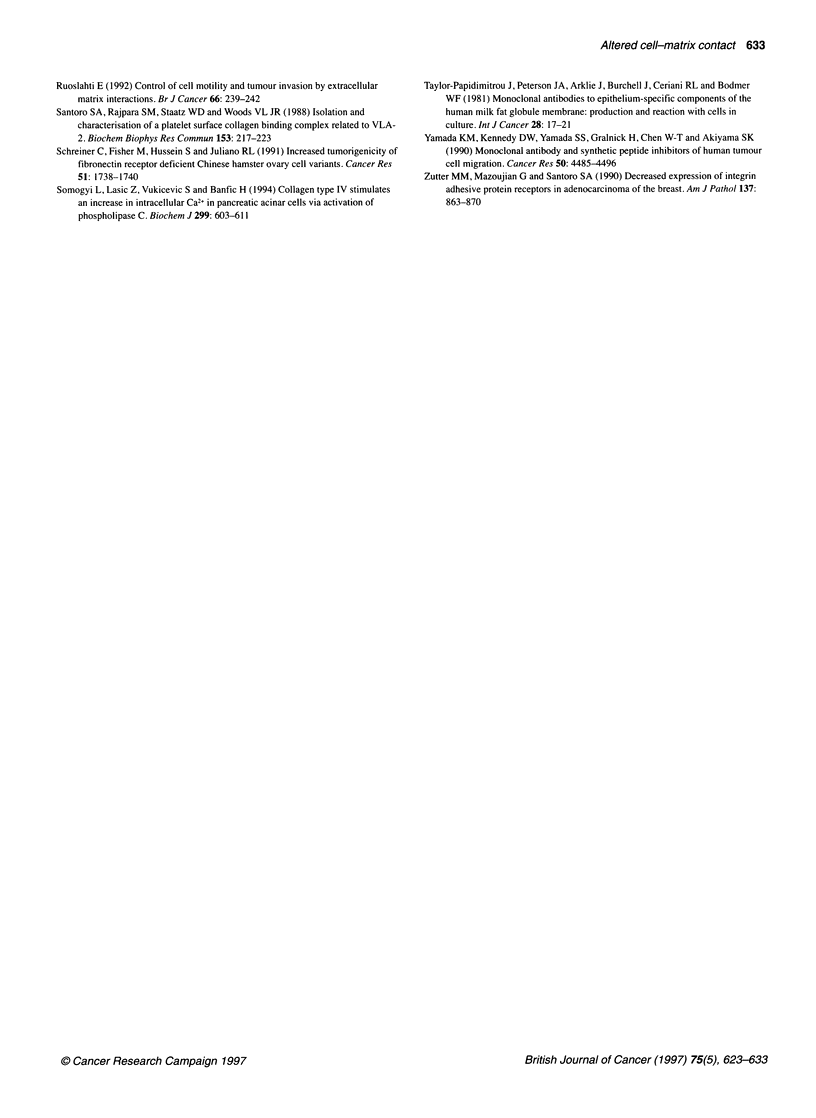# Altered cell-matrix contact: a prerequisite for breast cancer metastasis?

**DOI:** 10.1038/bjc.1997.113

**Published:** 1997

**Authors:** G. P. Gui, J. R. Puddefoot, G. P. Vinson, C. A. Wells, R. Carpenter

**Affiliations:** Department of Surgery, St Bartholomew's Hospital, West Smithfield, London, UK.

## Abstract

The integrins are receptors that regulate interaction between epithelial cells and the extracellular matrix. Previous studies have shown that a reduction in the expression of the alpha2beta1, alpha3beta1, alpha6beta1, alpha(v)beta1 and alpha(v)beta5 integrins in primary breast cancer is associated with positive nodal status. In order to assess the functional significance of altered integrin expression, primary breast cancer cells were derived from individual patients with known tumour characteristics using immunomagnetic separation. Purified human fibronectin, vitronectin, laminin and type IV collagen were used to represent the principal extracellular matrix proteins in an in vitro adhesion assay. Primary breast cancer cells from lymph node-positive patients were significantly less adhesive to each of the matrix proteins studied (P<0.001, Mann-Whitney U-test). Matrix adhesion of primary breast cancer cells from node-negative patients was inhibited by appropriate integrin monoclonal antibodies (P<0.001, paired Wilcoxon test). Adhesion to fibronectin, vitronectin and laminin, but not type IV collagen, was influenced by the inhibitor arginine-glycine-aspartate, suggesting that breast cancer cell recognition of collagen IV is mediated through alternative epitopes. Weak matrix adhesion correlated with loss of integrin expression in tissue sections from corresponding patients assessed using immunohistochemistry. This study demonstrates a link between altered integrin expression and function in primary breast cancers predisposed to metastasize.


*British Journal of Cancer* (1997) **75**(5), 623–633
© 1997 Cancer Research Campaign

### Altered cell–matrix contact: a prerequisite for breast cancer metastasis?

**GPH Gui[1], JR Puddefoot[2], GP Vinson[2], CA Wells[3] and R Carpenter[1]**

[1]Department of Surgery, The Royal Hospitals Trust Breast Unit, St Bartholomew's Hospital, 2nd Floor King George V Block, West Smithfield, London EC1A 7BE; [2]Department of Biochemistry, Queen Mary and Westfield College, Mile End Road, London E1 4NS; [3]Department of Pathology, The Royal Hospitals Trust Breast Unit, St Bartholomew's Hospital, 2nd Floor King George V Block, West Smithfield, London ECIA 7BE, UK

**Summary** The integrins are receptors that regulate interaction between epithelial cells and the extracellular matrix. Previous studies have shown that a reduction in the expression of the $\alpha 2\beta 1$, $\alpha 3\beta 1$, $\alpha 6\beta 1$, $\alpha v\beta 1$ and $\alpha v\beta 5$ integrins in primary breast cancer is associated with positive nodal status. In order to assess the functional significance of altered integrin expression, primary breast cancer cells were derived from individual patients with known tumour characteristics using immunomagnetic separation. Purified human fibronectin, vitronectin, laminin and type IV collagen were used to represent the principal extracellular matrix proteins in an in vitro adhesion assay. Primary breast cancer cells from lymph node-positive patients were significantly less adhesive to each of the matrix proteins studied ($P$<0.001, Mann–Whitney $U$-test). Matrix adhesion of primary breast cancer cells from node-negative patients was inhibited by appropriate integrin monoclonal antibodies ($P$<0.001, paired Wilcoxon test). Adhesion to fibronectin, vitronectin and laminin, but not type IV collagen, was influenced by the inhibitor arginine–glycine–aspartate, suggesting that breast cancer cell recognition of collagen IV is mediated through alternative epitopes. Weak matrix adhesion correlated with loss of integrin expression in tissue sections from corresponding patients assessed using immunohistochemistry. This study demonstrates a link between altered integrin expression and function in primary breast cancers predisposed to metastasize.

**Keywords:** breast cancer; integrin; cell adhesion; extracellular matrix; immunomagnetic separation; tumour metastasis

Focal contact with the extracellular matrix is a fundamental mechanism by which cells initiate intracytoplasmic signalling in order to regulate attachment, migration, differentiation and growth (Burridge et al, 1988). The anatomical basis for cell–matrix recognition is a group of transmembrane receptors called the cell adhesion molecules that include the integrins, cadherins, selectins and the immunoglobulin superfamily (Albelda and Buck, 1990). The integrins are the principal receptors that facilitate cellular interaction to the extracellular matrix through binding to specific sites in fibronectin, vitronectin, laminin and collagen (Hynes, 1992). The integrins consist of varying $\alpha$ and $\beta$ subunits that associate to form heterodimers. Fourteen $\alpha$ and eight $\beta$ subunits have been identified that associate to form 20 known integrins, each categorized into subfamilies based upon the common $\beta$ subunit (Hynes, 1987). Each integrin heterodimer bears a receptor site towards the amino terminal in the extracellular domain. The carboxyl end is contained within the cell and provides a link to the cytoskeleton via the cytosolic proteins talin, vinculin and actin (Horwitz et al, 1986; Otey et al, 1993). Intracellular signalling following ligation of integrin receptors is known to involve phosphorylation pathways (Kornberg et al, 1992). Current attention has focused on a novel 125 kDa protein called focal adhesion kinase $pp125^{fak}$ (Burridge et al, 1992). Integrins signal into the cell, as conventional receptors, but, in addition, are also able to transmit information from within the cell to the matrix as well as to other cells via a mechanism termed inside-out signalling (Hynes, 1992). The versatile nature of integrin function makes these receptors a critical point of study when cell–matrix interactions are considered.

Several unique peptide epitopes have been identified as recognition sequences for integrin receptors. The best characterized of these is the tripeptide arginine-glycine-aspartate, abbreviated to RGD (Humphries, 1990). While RGD may be used as a general inhibitor of integrin function, specific receptor–ligand interaction may be investigated using monoclonal antibodies raised against the component subunits of each heterodimer (Yamada et al, 1990). Altered cell–matrix interaction is an essential prerequisite step in the metastatic cascade (Hart and Saini, 1992). Reduced integrin expression has been described in primary breast cancer cells compared with their benign counterparts (Zutter et al, 1990; Pignatelli et al, 1991; Koukoulis et al, 1991), and altered function may be related to the presence of axillary nodal metastasis (Gui et al, 1995*a*). We have previously described a method of isolating live human breast cancer cells from individual patients for use in short term in vitro studies of adhesion to laminin (Gui et al, 1995*b*). The use of these patient-derived cells is of obvious benefit compared with propagated cell lines in that it allows the study of breast cancers in women with known pathological characteristics and nodal status. The aim of this study was to evaluate cell–matrix adhesion in tumour progression. Fibronectin and vitronectin were used to assess cell adhesion to the interstitial matrix, while laminin and type IV collagen were used to represent the basement membrane. Our hypothesis was that human primary breast cancer cells that had lost integrin expression were less adhesive and therefore predisposed to metastasize.

*Received 7 March 1996*
*Revised 7 August 1996*
*Accepted 26 September 1996*

*Correspondence to:* R Carpenter

Part of this work was presented to the Surgical Research Society in Glasgow on 6 July 1994

### MATERIALS AND METHODS

### Patients

Tumour samples were obtained from 41 patients with stage I or II invasive ductal breast cancer treated primarily by surgery at the Royal Hospitals Trust Breast Unit at St Bartholomew's. All patients were offered either a segmental or simple mastectomy with formal axillary dissection to evaluate nodal status. Routine staging investigations included a chest radiograph, serum liver enzyme biochemistry and corrected calcium. To exclude distant metastasis, liver ultrasonography and $^{99}$technetium radioisotope bone scans were performed when clinically indicated.

**Table 1** Tumour characteristics of patients studied

| | Node-negative ($n$ = 20) | Node-positive ($n$ = 21) |
|---|---|---|
| Mean patient age (range) years | 56.8(33–84) | 57.2(37–85) |
| Mean tumour size (range) cm | 2.4 (1.0–4.0) | 3.5 (1.0–9.0) |
| Grade (%) | | |
| I | $n$ = 4(20) | $n$ = 1(5) |
| II | $n$ = 7(35) | $n$ = 8(38) |
| III | $n$ = 9(45) | $n$ = 12(57) |
| Vascular invasion present (%) | $n$ = 1(5) | $n$ = 14(67) |
| Mean number of involved nodes (range) | 0 | 5.2(1–25) |
| Mean number of nodes obtained (range) | 13.3(5–26) | 12.4(3–26) |

### Inhibitory peptides and integrin monoclonal antibodies

The active inhibitor of integrin function, RGD, and the inactive tripeptide, arginine-glycine-glutamic acid (RGE), were obtained from Sigma Chemical Company, Poole, Dorset, UK. Integrin monoclonal antibodies against the α2 (P1E6), α3 (P1B5), β1 (MAb13), β3 (7F12), β5 (P1F6) (all from Becton-Dickinson, Oxford, UK) and α6 subunits (CLB-701) (Chemicon, London, UK) were used to evaluate integrin expression by immunohistochemistry, and cell–matrix adhesive function in the in vitro assay.

### Immunomagnetic separation

The method of isolating breast cancer cells using immunomagnetic separation was modified from that of Motoyasu et al (1989) for bile duct epithelium and has been described in detail in our previous work (Gui et al, 1995*b*). Briefly, fresh sterile breast cancer samples were transported in Dulbecco's modified Eagle medium (DMEM; Sigma) containing 4.5 g $l^{-1}$ glucose, 10% fetal bovine serum, 4 mmol $l^{-1}$ L-glutamine, 2 mmol $l^{-1}$ sodium pyruvate, 10 ml $l^{-1}$ non-essential amino acids, 100 U $ml^{-1}$ penicillin, 100 μg $ml^{-1}$ streptomycin, 5 μg $ml^{-1}$ fungizone and 50 U $ml^{-1}$ polymyxin B (ICN Biomedicals, Thame, UK). Each breast cancer segment was disaggregated in collagenase (Gibco BRL, Paisley, UK) at 5 units $mg^{-1}$ tissue in DMEM containing 5% fetal bovine serum, 5 μg $ml^{-1}$ bovine insulin, 100 U $ml^{-1}$ penicillin, 100 μg $ml^{-1}$ streptomycin, 5 μg $ml^{-1}$ fungizone and 50 U $ml^{-1}$ polymyxin B on a vertical rotator at 37°C overnight. Cells were recovered by centrifugation at 900 r.p.m. and viability assessed using trypan blue exclusion (Sigma). Dynabeads M-450 (Dynal UK, Wirral, Merseyside, UK), precoated with rat anti-mouse IgG2a, were incubated with antibody to epithelial membrane antigen (EMA) (Taylor-Papadimitrou et al, 1981) (Dako, High Wycombe, Bucks, UK) to target breast cancer cells in the disaggregated suspension. The assay was performed at 4°C to prevent non-specific adhesion. The beads were detached by a combination of immunological methods (Rasmussen et al, 1992) using Detach-a-Bead (Dynal UK) and mechanical procedures (Gui et al, 1995*b*).

Breast cancer cell clusters separated by the Magnetic Particle Container (Dynal UK) were dispersed into individual cells using 0.2% trypsin (ICN Biomedicals). The epithelial origin of the purified cell suspension was established by positive staining to EMA and the anti-human cytokeratin, CAM5.2, in conjunction with negative staining to the common leucocyte antigen CD45 and fibroblast markers (all from Dako UK). The malignant nature of the isolated cells was confirmed morphologically. Tissue samples were discarded, if adjacent cryostat sections contained recognizable benign breast cells. The reliability of immunomagnetic separation as a method of producing breast cancer cell isolates and data on cell survival times have been established previously (Gui et al, 1995*b*).

### Histology and immunochemistry

Routine tumour and lymph node pathology was assessed on 3-μm paraffin-embedded sections stained with haematoxylin and eosin. All negative lymph nodes were, in addition, stained with CAM5.2 (Becton-Dickinson) to exclude occult metastasis. Immunohistochemistry was performed on 5-μm primary breast cancer cryostat sections using the avidin–biotin–peroxidase complex technique (Hsu et al, 1981). Appropriate biotinylated antibodies and streptavidin peroxidase-conjugated antibodies (Dako UK) were used as secondary and tertiary layers respectively. All slides stained using immunohistochemistry were double read by two independent observers and scored as negative (–), weak (+), moderate (++) or strong (+++). Equivocal tumour cell staining was considered to be negative. Preservation of the integrin receptors after enzymatic disaggregation was confirmed by immunocytochemistry in cell smears snap frozen in liquid nitrogen.

### In vitro adhesion assay

In preliminary studies, flat-bottomed 96-well plates were coated with graded concentrations (0.1–1000 μg $ml^{-1}$) of each of purified human fibronectin, vitronectin, laminin (Collaborative Biomedical Products, Oxford, UK) and type IV collagen (Sigma). These experiments demonstrated that maximal cell adhesion to each protein was achieved after a 4-h incubation period at matrix concentrations of 100 μg $ml^{-1}$, and these parameters were selected as standard conditions for subsequent studies. Survival studies of primary breast cancer cell isolates based on trypan blue exclusion had shown viability in wells coated with these matrix proteins of up to 12 h after completion of immunomagnetic separation.

In the experiments described in this paper, all wells were rehydrated with 100 μg $ml^{-1}$ bovine serum albumin before use. The breast cancer cell suspension was diluted to contain 10 000 cells per ml in serum-free DMEM, and aliquots of 50 μl containing 500 cells were added to each well. Graded amounts of RGD, RGE (0–300 μg $ml^{-1}$) or integrin monoclonal antibodies (0–150 μg $ml^{-1}$) were used to inhibit cell–matrix adhesion. All experiments were conducted in triplicate at 37°C in a humidified carbon dioxide incubator. After incubation for 4 h, wells were washed and numbers of adherent cells estimated using spectrophotometric absorbance following uptake of 3-[4,5]dimethylthiazol-2-yl-2,5

© Cancer Research Campaign 1997

**Figure 1** Differences in primary breast cancer cell–matrix adhesion with nodal status. Cell adhesion was assessed to matrices of purified human (**A**) fibronectin, (**B**) vitronectin, (**C**) laminin and (**D**) type IV collagen, at concentrations of 100 μg ml$^{-1}$. Each box and whisker plot is represented by the median value (within the box) bounded by the 25th and 75th centiles (short edges of the box), while the bars show the 10th and 90th centiles. Individual observations lying outside this range are represented by circles. This convention is maintained in Figures 3 and 5. LN–, lymph node negative; LN+, lymph node positive; *P*, *P*-values, Mann–Whitney *U*-test

© Cancer Research Campaign 1997

**Figure 2** Inhibition of primary breast cancer cell adhesion by varying RGD concentrations to (**A**) fibronectin, (**B**) vitronectin, (**C**) laminin and (**D**) type IV collagen, each matrix at concentrations of 100 μg $ml^{-1}$. Primary breast cancer cells from integrin-positive patients were applied to triplicate wells. The bars represent the standard error of the mean diphenyltetrazolium bromide (MTT) by live cells (Carmichael et al, 1987) (Sigma).

### Statistical analysis

Cell–matrix adhesion between primary breast cancer cells derived from node-positive and node-negative women to each of fibronectin, vitronectin, laminin and type IV collagen was analysed using the Mann–Whitney *U*-test. Differences in response of patient-derived cell–matrix adhesion to inhibitors of integrin function for a given ligand were assessed as paired data by the Wilcoxon signed rank test. Good cell adhesion was defined as attachment of more than 50% of cells in triplicate studies, while attachment of less than 50% was considered poor adhesion. In order to facilitate statistical analysis by avoiding small data sets, integrin expression was dichotomized into positive and negative staining by combining the weak (+), moderate (++) and strong (+++) categories of positive expression. Integrin expression and function were compared using Fisher's exact test.

### RESULTS

### Variation in primary breast cancer cell–matrix adhesion with nodal status

The patient sample and tumour characteristics of the women studied are shown in Table 1. Owing to limitations on cell quantity required by these experiments, the actual number of patients studied for each matrix protein were: fibronectin $n = 40$ [19 lymph node-negative (LN–) and 21 lymph node-positive (LN+)], vitronectin $n = 40$ (20 LN–, 20 LN +), laminin $n = 39$ (20 LN–, 19 LN +) and type IV collagen $n = 39$ (19 LN–, 20 LN+).

© Cancer Research Campaign 1997

**Figure 3** Differential inhibition of primary breast cancer cell–matrix adhesion by RGD (150 μg ml$^{-1}$) with nodal status to (**A**) fibronectin, (**B**) vitronectin, (**C**) laminin and (**D**) type IV collagen, each matrix protein at concentrations of 100 μg ml$^{-1}$. See legend to Figure 1 for the convention applied to box and whisker plots. RGD, arginine-glycine-aspartate; RGE, arginine-glycine-glutamic acid (control); *P*, *P*-values, paired Wilcoxon test

Variation in adhesion of primary breast cancer cells with nodal status is shown in Figure 1. Adhesion of primary breast cancer cells from node-positive women to each of fibronectin, vitronectin, laminin and type IV collagen was significantly less than that of node-negative patients ($P$<0.001, Mann–Whitney $U$-test). Primary breast cancer cells that were predisposed to metastasize were thus less adherent to the extracellular matrix than primary breast cancer cells from lymph node-negative patients. To show that poor adhesion of primary breast cancer cells from lymph node-positive patients was not caused by inadequate incubation, cells from six

© Cancer Research Campaign 1997

**Figure 4** Inhibition of primary breast cancer cell adhesion by varying concentrations of integrin monoclonal antibodies to (**A**) fibronectin, (**B**) vitronectin, (**C**) laminin and (**D**) type IV collagen, each matrix at concentrations of 100 μg ml$^{-1}$. Integrin-positive breast cancer cells were applied to matrix protein-coated wells in triplicate. The bars represent the standard error of the mean. (—○—)PIE6; (-△-)CLB-701; (—◇—)7F12; (-□-)PIB5; (-▽-)MAb 13; (-O-)PIF6

node-positive women were incubated for 8 h at 100 μg ml$^{-1}$, and for 4 h at 1000 μg ml$^{-1}$ of each matrix protein with no demonstrable increase in cell adhesion. The inhibitory tripeptide RGD and specific integrin monoclonal antibodies were used to show that this difference in cell–matrix adhesion between lymph node-positive and node-negative women was integrin mediated.

### Variation in RGD inhibition of breast cancer cell adhesion with nodal status

Differences in the inhibitory effects of RGD on primary breast cancer cell adhesion to fibronectin, vitronectin, laminin and type IV collagen with nodal status were investigated at concentrations of 100 μg ml$^{-1}$ of each matrix protein. The dose–response of increased inhibition of cell–matrix adhesion by greater doses of RGD is shown in Figure 2, using primary breast cancer cells obtained from six consecutive integrin-positive patients. The inactive tripeptide RGE was used as a control. Maximal inhibition of cell–matrix adhesion was achieved using 150 μg ml$^{-1}$ RGD. This concentration of RGD was therefore used to evaluate differential inhibition of primary breast cancer cell adhesion to each matrix protein by RGD (Figure 3). Cell–matrix adhesion of primary breast cancer cells derived from lymph node-negative patients to fibronectin, vitronectin and laminin were significantly inhibited by

© Cancer Research Campaign 1997

**Figure 5** Differences in inhibition of primary breast cancer cell–matrix adhesion with nodal status by integrin antibodies at concentrations of 75 μg ml⁻¹ to (**A**) fibronectin, (**B**) vitronectin, (**C**) laminin and (**D**) collagen IV. Each matrix protein was coated at 100 μg ml⁻¹. See legend to Figure 1 for the convention applied to the box and whisker plots. *P*, *P*-values, paired Wilcoxon test

© Cancer Research Campaign 1997

**Table 2** Comparison of integrin expression and adhesive function to each matrix protein

| | Cell–matrix adhesion (expressed as % of total number of plated cells) | | | | | | | | | | | |
|---|---|---|---|---|---|---|---|---|---|---|---|---|
| | Fibronectin | | | Vitronectin | | | Laminin | | | Type IV collagen | | |
| | >50% | ≤50% | *P* | >50% | ≤50% | *P* | >50% | ≤50% | *P* | >50% | ≤50% | *P* |
| Integrin expression | | | | | | | | | | | | |
| α2 | | | | | | | | | | | | |
| +++ | NA | | | NA | | | 1 | 0 | | 1 | 0 | |
| ++ | | | | | | | 9 | 2 | | 8 | 3 | |
| + | | | | | | | 5 | 1 | | 4 | 1 | |
| 0 | | | | | | | 3 | 16 | < 0.001 | 3 | 16 | < 0.001 |
| Missing data | | | | | | | 2 | 0 | | 3 | 0 | |
| α3 | | | | | | | | | | | | |
| +++ | 0 | 0 | | NA | | | 0 | 0 | | 0 | 0 | |
| ++ | 4 | 2 | | | | | 6 | 1 | | 4 | 2 | |
| + | 7 | 2 | | | | | 7 | 2 | | 7 | 1 | |
| 0 | 8 | 17 | 0.011 | | | | 6 | 16 | 0.001 | 5 | 17 | 0.001 |
| Missing data | 0 | 0 | | | | | 1 | 0 | | 3 | 0 | |
| α6 | | | | | | | | | | | | |
| +++ | NA | | | NA | | | 2 | 0 | | NA | | |
| ++ | | | | | | | 7 | 2 | | | | |
| + | | | | | | | 6 | 2 | | | | |
| 0 | | | | | | | 4 | 15 | <0.001 | | | |
| Missing data | | | | | | | 1 | 0 | | | | |
| β1 | | | | | | | | | | | | |
| +++ | 3 | 0 | | 3 | 0 | | 3 | 0 | | 3 | 0 | |
| ++ | 6 | 4 | | 7 | 5 | | 12 | 1 | | 7 | 4 | |
| + | 6 | 2 | | 6 | 2 | | 2 | 3 | | 4 | 2 | |
| 0 | 4 | 15 | 0.002 | 3 | 13 | 0.002 | 2 | 14 | <0.001 | 3 | 14 | 0.002 |
| Missing data | 0 | 0 | | 1 | 0 | | 1 | 1 | | 2 | 0 | |
| β5 | | | | | | | | | | | | |
| +++ | 0 | 0 | | 0 | 0 | | N/A | | | NA | | |
| ++ | 0 | 1 | | 0 | 1 | | | | | | | |
| + | 11 | 3 | | 10 | 2 | | | | | | | |
| 0 | 7 | 15 | 0.013 | 6 | 17 | 0.004 | | | | | | |
| Missing data | 1 | 2 | | 4 | 1 | | | | | | | |

Integrin expression was scored as strong (+++), moderate (++), weak (+), or negative staining (0) by two independent observers with consensus. Integrin function was expressed as the mean percentage of cell adhesion plated in triplicate wells. Strong and weak adhesion was considered to be greater or less than 50% cell–matrix adhesion respectively. Missing data arising from insufficient numbers of cells for an experimental set is shown wherever appropriate. Integrin subunits that were not applicable (NA) for a given matrix protein were not included. For non-parametric analysis, data for integrin expression were categorized into positive and negative by combining the strong, moderate and weak staining groups. *P*-values were derived using Fisher's exact test.

RGD, but adhesion to type IV collagen was unaffected by this tripeptide motif. Primary breast cancer cell adhesion to type IV collagen may, therefore, be mediated through alternative peptide recognition sequences. The control tripeptide RGE had no effect on breast cancer cell adhesion to the extracellular matrix. Primary breast cancer cells derived from lymph node-positive patients were unaffected by RGD, although any effect on cell–matrix adhesion may have been masked by weak adhesion even in the absence of inhibitory peptides.

### Inhibition of primary breast cancer cell adhesion by integrin antibodies

Relevant antibodies to the integrins were tested against each of fibronectin, vitronectin, laminin and type IV collagen in primary breast cancer cells obtained from six consecutive integrin-positive tumours (Figure 4). Inhibition of cell–matrix adhesion by appropriate integrin monoclonal antibodies was achieved in a dose-dependent manner. Maximal inhibition of attachment was achieved at concentrations of 75 $\mu g\ ml^{-1}$ of each integrin monoclonal antibody for all the matrix proteins studied. P1F6, an inhibitory antibody against αvβ5, had no effect on primary breast cancer cell adhesion to fibronectin, even at a concentration of 150 $\mu g\ ml^{-1}$. This suggests that αvβ5 expressed in human breast cancer cells is not a receptor for fibronectin. Each monoclonal antibody was used at concentrations of 75 $\mu g\ ml^{-1}$ to assess variation in the inhibition of cell–matrix adhesion with nodal status at 100 $\mu g\ ml^{-1}$ of each matrix protein (Figure 5 A–D). Primary breast cancer cells derived from lymph node-negative women demonstrated good adhesion to the extracellular matrix and were inhibited by appropriate integrin monoclonal antibodies. Cell adhesion to fibronectin was inhibited by antibodies to the α3 and β1 subunits; vitronectin by β1 and β5; laminin by α2, α3, α6 and β1; and type IV collagen by α2, α3 and β1.

Primary breast cancer cells derived from lymph node-positive patients were poorly adherent to control wells that were incubated in the absence of inhibitory monoclonal antibodies. This intrinsic weak cell adhesion profile may conceal any potential effect of integrin monoclonal antibodies on cell–matrix interaction.

 © Cancer Research Campaign 1997

### Integrin expression and function in primary breast cancer cells

To compare integrin expression and function, the following integrin subunits were used: α3, β1 and β5 for fibronectin; β1 and β5 for vitronectin; α2, α3, α6 and β1 for laminin; and α2, α3 and β1 for collagen type IV. A significant relationship between integrin expression and cell–matrix adhesive function was demonstrated using Fisher's exact test (Table 2). A proportion of tumours was not assessed for both integrin expression and function for each subunit because of insufficient numbers of primary breast cancer cells after immunomagnetic separation. Missing variables were few and these are listed for each matrix protein in Table 2. Positive integrin expression in primary breast cancer cells assessed using immunohistochemistry was associated with good adhesion (> 50%) to each of the matrix proteins studied, while the converse was true for breast cancer cells with negative integrin expression. The few patients that demonstrated strong integrin expression (+++) all displayed strong adhesion in excess of 70%. This study demonstrates for the first time a relationship between integrin receptor expression and function in patient-derived human breast cancer cells.

### DISCUSSION

Interaction between cancer cells and the extracellular matrix is known to influence tumour outcome (Ruoslahti, 1992) and may provide a mechanism by which certain tumour types are prone to metastasize. The cell adhesion molecules could be responsible for this phenomenon with variable expression and function of these receptors. Cell adhesion receptor function might be required for cancer cell interaction with the endothelium of lymphovascular channels and migration through the extracellular matrix at primary as well as secondary sites (Aznavoorian et al, 1990). On the other hand, down-regulation of cell adhesion molecule function might be expected in order for cancer cells to detach from the primary mass in the metastatic process. High integrin function is a recognized stop signal in the control of cell migration in embryogenesis (Duband et al, 1986). In cancer, reduced adhesion may result in less sticky tumour cells able to move unhindered in the extracellular matrix, thus predisposed to metastasize. Experimental work on the tumour biology of metastasis has conventionally used cell lines in in vitro and animal models. The relationship between these models and the human situation is unreliable and attempts to extrapolate the data from such experiments to the clinical context is difficult. We, therefore, addressed this problem by the development of a method of isolating human primary breast cancer cells for use in short-term in vitro studies.

The role of the integrins in modulating matrix adhesion in primary breast cancer cells from individual patients with known tumour characteristics has not been investigated previously. We and others have identified specific integrin subunits that are down-regulated in breast cancer (Zutter et al, 1990; Pignatelli et al, 1991; Koukoulis et al, 1991; Gui et al, 1995*a*), leading to the use of specific monoclonal antibodies to study function. Our results indicate that primary breast cancer cells from node-negative women were significantly more adherent to each of fibronectin, vitronectin, laminin and type IV collagen compared with primary breast cancer cells derived from node-positive patients (Figure 1). This was shown to be mediated, at least in part, by integrin interaction using the inhibitory tripeptide RGD (Figure 3). Inhibition of cell adhesion by RGD reflects the recognition by integrin receptors of this motif in the extracellular matrix. In node-negative women, breast cancer cell adhesion to fibronectin, vitronectin and laminin was significantly inhibited by RGD. The apparent inactivity of RGD in mediating cell binding to collagen IV probably results from the relative unimportance of this motif as a recognition sequence in cell–matrix adhesion to this ligand. RGD has been shown to have an important role in the modulation of other homeostatic cellular mechanisms, such as the regulation of cytoplasmic calcium levels in pancreatic acini by collagen type IV (Somogyi et al, 1994). We were unable to demonstrate any RGD-mediated effect on cell adhesion to type IV collagen in this study.

The specific influence of each integrin receptor was then further assessed using monoclonal antibodies directed against individual subunits. Primary breast cancer cell–matrix adhesion of node-negative women was significantly reduced compared with control wells using the paired Wilcoxon test (Figure 5). Inhibition of breast cancer cell adhesion by monoclonal antibodies in node-negative women was consistent with previous knowledge of integrin receptor–ligand interactions (Hynes, 1992). Thus, cell adhesion to fibronectin was inhibited by antibodies to the α3β1 integrin; vitronectin by αvβ1 and αvβ5; laminin by α2β1, α3β1 and α6β1; and type IV collagen by α2β1 and α3β1. There were two interesting exceptions: P1F6 (anti-β5) did not inhibit cell adhesion to fibronectin and 7F12 (anti-β3) failed to inhibit attachment to vitronectin (Figure 5A and B). From this, we can conclude that, in human primary breast cancer, αvβ5 is a vitronectin but not a fibronectin receptor, although it has been shown to recognize both proteins in other cell types (Albelda and Buck, 1990). The αvβ3 integrin did not influence cell adhesion in breast cancer cells and this supports our previous finding that altered expression of β3 is not a determinant of nodal status (Gui et al, 1995*a*).

Cell–matrix adhesion of primary breast cancers from node-positive women demonstrated poor adhesion and was not affected by inhibitory peptides or antibodies, providing functional evidence that these cells had lost integrin expression. The differences in integrin-mediated primary breast cancer cell–matrix adhesion with nodal status were shown to be unrelated to extreme incubation times, matrix protein and inhibitor concentrations. These data support the hypothesis that primary breast cancer cells that have lost integrin function are predisposed to metastasize.

Immunochemistry may be criticized as a method of investigating the multistep process of tumour metastasis, as it provides only a snapshot of a dynamic process. Expression of cell surface receptors demonstrated by special stains may not relate to functional activity. In this study, we have shown a significant relationship between integrin expression and adhesive function (Table 2). However, a proportion of primary breast cancer cells with weak (+) or moderate (++) integrin expression demonstrated poor adhesion to the extracellular matrix, suggesting some receptors are only partially functional or non-functional. The discrepancy between integrin expression and function observed in a proportion of cases may also have arisen because of the complex relationship between the integrins and their ligands. As an example, consider the α2β1 and α3β1 integrins that are receptors to both laminin (Languino et al, 1989) and collagen (Santoro et al, 1988). Positive integrin expression of α2β1 and α3β1 measured using immunochemistry might have been interpreted erroneously as an indicator of a functional relationship with either protein. Another reason for this observation may be the non-availability of functional monoclonal antibodies against the α1, αv, β6 and β8 subunits. The αv subunit

© Cancer Research Campaign 1997 

forms distinct integrin heterodimers with both these newly discovered β6 and β8 subunits, as well as β1, β3 and β5. The role of αvβ6 and αvβ8 integrins in breast cancer progression therefore remains to be clarified. The mechanism by which integrin receptors are able to recognize multiple ligands is as yet uncertain. Integrin receptor heterodimers that bind to identical proteins are known to recognize specific epitopes that are spatially distinct on the matrix macromolecule (Humphries et al, 1987; Hall et al, 1990).

Integrin receptors undergo conformational change between an active and inactive state (Hynes, 1992), and receptor activation may be influenced by a variety of factors. Modulation of integrin receptor function might be regulated by direct ligation of the receptor itself (outside-in signalling), a phenomenon associated with receptor clustering (Philips et al, 1988). However, integrin expression is mainly thought to be regulated by inside-out signalling through a variety of tissue and soluble factors, including the cell adhesion molecule CD44 (Koopman et al, 1990), c-erbB-2 (D'Souza et al, 1993), and the interleukins IL-12 (Rabinowich et al, 1993*a*) and IL-6 (Rabinowich et al, 1993*b*). Integrin expression is also dependent on the divalent cations calcium and manganese (Humphries, 1990). The regulation of integrin receptor expression and function in tumour progression is currently being investigated in our laboratory.

The cellular effects modulated by integrin expression in tumour invasion may only be empirically related to cell–matrix adhesion. Loss of integrin expression may predispose to tumour progression by the loss of regulatory control over growth and differentiation. In in vitro studies, transfection of Chinese hamster ovary cells with the cDNA for the α5β1 fibronectin receptor inhibits the ability of these cells to grow and establish tumours in nude mice (Giancotti and Ruoslahti, 1990). Cancer cells with decreased integrin expression may thus display greater invasive potential (Schreiner et al, 1991).

The biology of tumour metastasis is still not well understood. We have shown that loss of integrin expression and function in primary breast cancer cells are related to reduced matrix adhesion in the presence of axillary nodal metastasis. Altered integrin-mediated adhesion of cancer cells to the extracellular matrix is a critical factor in the metastatic cascade. The integrins may, thus, be of clinical value as predictors of nodal status in patients with breast cancer at the time of symptomatic presentation. The prognostic significance of integrin expression in determining disease-free interval and overall survival in breast cancer progression remains to be defined.

### ACKNOWLEDGEMENTS

We are grateful to the Joint Research Board of Trustees of St Bartholomew's Hospital and the South Essex Medical Education and Research Trust for providing funding for this project.

### REFERENCES

Albelda SM and Buck CA (1990) Integrins and other cell adhesion molecules. *FASEBJ* **4**: 2868–2880

Aznavoorian S, Stracke ML, Krutzsch H, Schiffmann E and Liotta LA (1990) Signal transduction for chemotaxis and haptotaxis by matrix molecules in tumor cells. *J Cell Biol* **110**: 1427–1438

Burridge K, Fath K, Kelly T, Nuckolis B and Turner C (1988) Focal adhesions: transmembrane junctions between the extracellular matrix and the cytoskeleton. *Annu Rev Cell Biol* **4**: 487–525

Burridge K, Turner CE and Romer L (1992) Tyrosine phosphorylation of paxillin and pp125FAK accompanies cell adhesion to extracellular matrix: a role in cytoskeletal assembly. *J Cell Biol* **119**: 893–903

Carmichael J, Degraff WG, Gazdar AF, Minna JD and Mitchell JB (1987) Evaluation of a tetrazolium-based semi-automated colorimetric assay: assessment of radiosensitivity. *Cancer Res* **47**: 943–946

D'souza B, Berdichevsky F, Kyprianou N and Taylor-Papadimitrou J (1993) Collagen-induced morphogenesis and expression of the α2-integrin subunit is inhibited in c-erbB2-transfected human mammary epithelial cells. *Oncogene* **8**: 1797–1806

Duband J-L, Rocher S, Chen W-T, Yamada KM and Thiery JP (1986) Cell adhesion and migration in the early vertebrate embryo: location and possible role of the putative fibronectin receptor complex. *J Cell Biol* **102**: 160–178

Giancotti FG and Ruoslahti E (1990) Elevated levels of the α5β1 fibronectin receptor suppress the transformed phenotype of Chinese hamster ovary cells. *Cell* **60**: 849–859

Gui GPH, Wells CA, Browne PD, Yeomans P, Jordan S, Puddefoot JR, Vinson GP and Carpenter R (1995*a*) Integrin expression in primary breast cancer and its relation to axillary nodal status. *Surgery* **117**: 102–108

Gui GPH, Puddefoot JR, Vinson GP, Wells CA and Carpenter R (1995*b*) Modulation of VLA-2 laminin receptor in breast cancer metastasis. *Surgery* **118**: 245–250

Hall DE, Reichardt LF, Crowley E, Holley B, Moezzi H, Sonnenberg A and Damsky CH (1990) The α1/β1 and α/6β1 integrin heterodimers mediate cell attachment to distinct sites on laminin. *J Cell Biol* **110**: 2175–2184

Hart IR and Saini A (1992) Biology of tumour metastasis. *Lancet* **339**: 1453–1457

Horwitz A, Duggan K, Buck C, Beckerle MC and Burridge K (1986) Interaction of plasma membrane fibronectin receptors with talin – a transmembrane linkage. *Nature* **320**: 531–533

Hsu SM, Raine L and Fanger H (1981) Use of avidin–biotin–peroxidase complex (ABC) in immunoperoxidase techniques: a comparison between ABC and unlabelled antibody (PAP) procedures. *J Histochem Cytochem* **29**: 577–580

Humphries MJ (1990) The molecular basis and specificity of integrin-ligand interactions. *J Cell Sci* **97**: 585–592

Humphries MJ, Komoriya A, Akiyama SK, Olden K and Yamada KM (1987) Identification of two distinct regions of the type III connecting segment of human plasma fibronectin that promote cell type-specific adhesion. *J Biol Chem* **262**: 6886–6892

Hynes RO (1987) Integrins: a family of cell surface receptors. *Cell* **48**: 549–554

Hynes RO (1992) Integrins: versatility, modulation, and signalling in cell adhesion. *Cell* **69**: 11–25

Koopman G, Van Koogh Y, DE Graaf M, Meyer CJ, Figdor CG and Pals ST (1990) Triggering of the CD44 antigen in lymphocytes promotes T cell adhesion through the LFA-1 pathways. *J Immunol* **145**: 3589–3593

Kornberg L, Earp HS, Parsons JT, Schaller M and Juliano RL (1992) Cell adhesion of integrin clustering increases phosphorylation of a focal adhesion associated tyrosine kinase. *J Biol Chem* **267**: 23439–23442

Koukoulis GK, Virtanen I, Korhonen M, Laitinen L, Quaranta V and Gould VE (1991) Immunohistochemical localisation of integrins in the normal, hyperplastic, and neoplastic breast. *Am J Pathol* **139**: 787–799

Languino LR, Gehlsen KR, Wayner EA, Carter WG, Engvall E and Ruoslahti E (1989) Endothelial cells use α2β1 integrin as a laminin receptor. *J Cell Biol* **109**: 2455–2462

Motoyasu I, Vroman B and Larusso NF (1989) Isolation and morphologic characterization of bile duct epithelial cells from normal rat liver. *Gastroenterology* **97**: 1236–1247

Otey CA, Vasquez GB, Burridge K and Erikson BW (1993) Mapping of the alpha-actinin binding site within the beta-1 integrin cytoplasmic domain. *J Biol Chem* **268**: 2193–2197

Phillips DR, Charo IF, Parise LV and Fitzgerald LA (1988) The platelet membrane glycoprotein IIb–IIIa complex. *Blood* **71**: 831–843

Pignatelli M, Hanby AM and Stamp GWH (1991) Low expression of β1, α1 and α3 subunits of VLA integrins in malignant mammary tumours. *J Pathol* **165**: 25–32

Rabinowich H, Herberman RB and Whiteside TL (1993*a*) Differential effects of IL12 and IL2 on expression and function of cellular adhesion molecules on purified human natural killer cells. *Cell Immunol* **152**: 481–498

Rabinowich H, Sedlmayr P, Herberman RB and Whiteside TL (1993*b*) Response of human NK cells to IL-6 alteration of the cell surface phenotype, adhesion to fibronectin and laminin, and tumour necrosis factor – alpha/beta secretion. *J Immunol* **150**: 4844–4855

Rasmussen A-M, Smeland EB, Erikstein BK, Caignault L and Funderud S (1992) A new method for detachment of Dynabeads from positively selected B lymphocytes. *J Immunol Methods* **146**: 195–202

© Cancer Research Campaign 1997

Ruoslahti E (1992) Control of cell motility and tumour invasion by extracellular matrix interactions. *Br J Cancer* **66**: 239–242

Santoro SA, Rajpara SM, Staatz WD and Woods VL JR (1988) Isolation and characterisation of a platelet surface collagen binding complex related to VLA-2. *Biochem Biophys Res Commun* **153**: 217–223

Schreiner C, Fisher M, Hussein S and Juliano RL (1991) Increased tumorigenicity of fibronectin receptor deficient Chinese hamster ovary cell variants. *Cancer Res* **51**: 1738–1740

Somogyi L, Lasic Z, Vukicevic S and Banfic H (1994) Collagen type IV stimulates an increase in intracellular $Ca^{2+}$ in pancreatic acinar cells via activation of phospholipase C. *Biochem J* **299**: 603–611

Taylor-Papidimitrou J, Peterson JA, Arklie J, Burchell J, Ceriani RL and Bodmer WF (1981) Monoclonal antibodies to epithelium-specific components of the human milk fat globule membrane: production and reaction with cells in culture. *Int J Cancer* **28**: 17–21

Yamada KM, Kennedy DW, Yamada SS, Gralnick H, Chen W-T and Akiyama SK (1990) Monoclonal antibody and synthetic peptide inhibitors of human tumour cell migration. *Cancer Res* **50**: 4485–4496

Zutter MM, Mazoujian G and Santoro SA (1990) Decreased expression of integrin adhesive protein receptors in adenocarcinoma of the breast. *Am J Pathol* **137**: 863–870

© Cancer Research Campaign 1997